# Monthly mini-dose rituximab for primary anti-PLA2R-positive membranous nephropathy: a personalized approach

**DOI:** 10.1186/s12882-023-03206-1

**Published:** 2023-05-26

**Authors:** Song Wang, Zhenling Deng, Yue Wang, Wenhan Bao, Sijia Zhou, Zhuan Cui, Danxia Zheng

**Affiliations:** Department of Nephrology, Peking University Third Hospital, No. 49 North Garden Road, 100191 Beijing, China

**Keywords:** Anti-phospholipase A2 receptor antibody, Membranous nephropathy, Nephrotic syndrome, Rituximab, Mini-dose

## Abstract

**Background:**

The currently recommended dose of rituximab for primary membranous nephropathy is as high as that for lymphoma. However, the clinical manifestations of membranous nephropathy vary widely. Therefore, achieving individualized treatment is a topic that needs to be explored. This study assessed the efficacy of monthly mini-dose rituximab monotherapy in patients with primary membranous nephropathy.

**Methods:**

This retrospective study included 32 patients with primary membranous nephropathy treated at Peking University Third Hospital between March 2019 and January 2023. All patients were anti-phospholipase A2 receptor (PLA2R) antibody-positive and received rituximab 100 mg intravenously monthly for at least 3 months without other immunosuppressive therapy. Rituximab infusions were sustained until either remission of the nephrotic syndrome or a minimum serum anti-PLA2R titer ˂ 2 RU/mL was achieved.

**Results:**

The baseline parameters included: proteinuria, 8.5 ± 3.6 g/day; serum albumin, 24.8 ± 3.4 g/L; and anti-PLA2R antibody, 160 (20–2659) RU/mL. B-cell depletion was achieved in 87.5% patients after the first dose of rituximab 100 mg and in 100% after the second equivalent dose. The median follow-up was 24 months (range 18–38). Twenty-seven (84%) patients achieved remission, with 11 (34%) patients achieving complete remission by last follow-up. The relapse-free survival from the last infusion was 13.5 months (range 3–27). Patients were stratified into the low-titer (< 150 RU/mL, *n* = 17) and high-titer groups (≥ 150 RU/mL, *n* = 15) based on the anti-PLA2R titer. Sex, age, urinary proteins, serum albumin, and estimated glomerular filtration rate at baseline did not differ significantly between the two groups. At 18 months, compared to the low-titer group, the rituximab dose (960 ± 387 vs 694 ± 270 mg, *p* = 0.030) was higher, while serum albumin (37.0 ± 5.4 vs 41.3 ± 5.4 g/L, *p* = 0.033) and the complete remission rate (13% vs 53%, *p* = 0.000) were both lower in the high-titer group.

**Conclusions:**

Monthly rituximab 100 mg appeared as a potential effective regimen for treating anti-PLA2R-associated primary membranous nephropathy with a low anti-PLA2R titer. The lower the anti-PLA2R titer, the lower the rituximab dose required to achieve remission.

**Trial registration:**

A retrospective study, registered at ChiCTR (ChiCTR2200057381) on March 10, 2022.

## Introduction

Primary membranous nephropathy (PMN) is among the most common causes of nephrotic syndrome (NS) in non-diabetic adults [1, 2]. Ten-year follow-up data from two independent trials demonstrate a 35%-40% rate of reaching kidney failure in patients treated conservatively, compared with an 8%-11% rate in patients treated with an alkylating agent/corticosteroid regimen [1]. Sustained massive proteinuria is an important risk factor for progression to end-stage renal disease (ESRD). Amelioration or complete inhibition of proteinuria can greatly retard the occurrence of ESRD in patients with PMN. Clinicians have always aimed to devise methods to derive the maximal clinical benefit from a given therapeutic modality with the minimal medical cost. To that end, clinicians may consider designing individualized treatment regimens for different patients according to disease severity, among other factors. Rituximab, a human/murine, chimeric anti-CD20 monoclonal antibody that induces rapid and long-term depletion of CD20 + B cells, has been used for the treatment of PMN since 2002. Studies have demonstrated its efficacy in inducing remission in approximately two-thirds of patients and its superior safety profile over cyclophosphamide or calcineurin inhibitors, after observation for more than 12 months [3–8].

Most studies that investigated the efficacy of rituximab for PMN utilized the same strategy as that for lymphoma, *i.e*., intravenous administration at a dose of 375 mg/m^2^/week for 4 consecutive weeks, or 1000 mg on days 1 and 15 [3–8]. As the number and activity of B lymphocytes in PMN are considerably lower than those in lymphoma, a larger dosing regimen is relatively expensive and can increase the risk of infections [9, 10]. This forms the rationale for the reduction in the rituximab dose to achieve the same therapeutic effect in PMN. In our clinical experience, we found that rituximab 100 mg could achieve B-cell depletion in most patients, which could be sustained for more than one month. We postulated that monthly intermittent dosing could maintain B-cell exhaustion, such that the criterion for immunological remission of PMN would be met [*i.e.*, the titer of anti-phospholipase A2 receptor (PLA2R) antibody, a specific antibody in PMN, would gradually become negative], and eventually result in clinical remission. Herein, we report the efficacy of rituximab 100 mg administered monthly to anti-PLA2R-positive PMN patients with NS.

## Methods

**Fig. 1:**
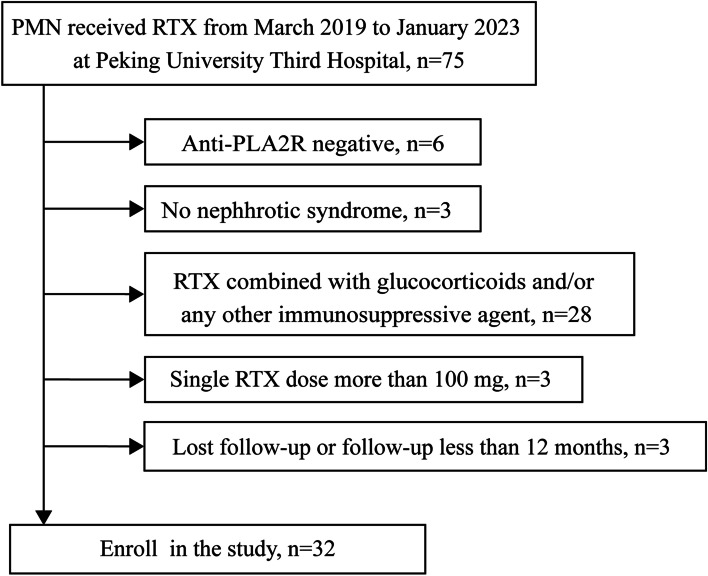
Flowchart of enrollment

Patients with PMN who received rituximab therapy between March 2019 and January 2023 were enrolled in the study. The inclusion criteria were as follows: (1) age more than 18 years, (2) PMN proven by biopsy, (3) NS defined as proteinuria ≥ 3.5 g/day and serum albumin (ALB) < 30 g/L with or without hyperlipidemia or edema, (4) estimated glomerular filtration rate (eGFR) ≥ 30 mL/min/1.73 m^2^, and (5) serum anti-PLA2R antibody > 20 RU/mL. The exclusion criteria were as follows: (1) secondary membranous nephropathy, (2) absence of NS at baseline, (3) concurrent use of glucocorticoids and/or any other immunosuppressive agent at baseline, (4) patients who received a single rituximab infusion of more than 100 mg, and (5) loss to follow-up or follow-up for less than 12 months. The flowchart of the study selection process is depicted in Fig. 1.

This retrospective study was approved by the Research Ethics Committee of Peking University Third Hospital Medical Science (M2022060) on March 2, 2022, and registered at ChiCTR (ChiCTR2200057381) on March 10, 2022. After receiving ethical approval, patients signed written informed consent at follow-up.

### Rituximab treatment protocol

The initial dose of rituximab 100 mg was administered by intravenous injection (in conjunction with dexamethasone 5 mg, paracetamol 0.6 g, and promethazine 5 mg). Subsequent doses of rituximab 100 mg were administered after an interval of 4 ± 2 weeks, until remission of NS or a minimum serum anti-PLA2R titer ˂ 2 RU/mL was achieved. Patients with infections were screened and excluded prior to each rituximab administration. Interruptions in the monthly rituximab regimen were allowed due to the outbreak of the coronavirus disease (COVID-19) epidemic and control policy in China that prevented patients from visiting the hospital, incidence of any infection, patients’ unwillingness, etc. The frequency of infusion was reduced in some patients after partial remission of NS.

Flow cytometry was used to determine CD19 + and CD20 + B-cell counts in peripheral blood. The serum anti-PLA2R titer was assessed using an enzyme-linked immunosorbent assay (ELISA) kit purchased from Euroimmune (Lubeck, Germany). A titer ≥ 20 RU/mL was defined as positive according to the manufacturer’s protocol, and a titer < 2 RU/mL was defined as negative according to literature [11]. The serum rituximab concentration was measured using an ELISA kit purchased from Abcam (Cambridge, MA, U.S.A.). The concentrations of serum creatinine, 24-h urinary protein, serum ALB, immunoglobulins, and complements were assessed routinely. The eGFR was calculated using the Chronic Kidney Disease Epidemiology Collaboration formula.

Complete remission (CR) was defined as a urinary protein excretion < 0.3 g/day, serum ALB ≥ 35 g/L, and stable renal function. Partial remission was defined as a 24-h urinary protein excretion between 0.3–3.5 g and at least 50% reduction from baseline, ALB ≥ 30 g/L, and stable renal function. Patients who did not meet the above-mentioned criteria were considered to be in non-remission. Relapse was defined as the resurgence of proteinuria > 3.5 g/day and/or ALB < 30 g/L during at least two consecutive visits in patients who had previously achieved a partial or complete response. B-cell depletion was defined as an absolute CD19 + cell count < 5/mm^3^in peripheral blood [12].

Statistical analysis was performed using SPSS software (SPSS 23.0. Armonk, NY: IBM Corp). Data were summarized as frequency (%), mean ± standard deviation (SD), or median (range), as appropriate. The baseline numerical variables and follow-up data were compared using a paired sample t-test. Comparisons between two groups were performed using an independent sample t-test. The two rates were compared using the chi-squared test. Statistical significance was set at *p* < 0.05.

## Results

A total of 32 patients met the selection criteria, of which 25 were men and 7 were women. The participants’ average age was 55 ± 15 years (range, 19–76). The median time from onset/recurrence of membranous nephropathy to rituximab therapy was 5 months (range 1–36). Twenty-four patients were naive of immunosuppressive therapy, and eight patients were previously treated with glucocorticoids and immunosuppressants. Six recurrent patients were treated with rituximab as first-line therapy. The other two patients received immunosuppressive agents for 3 months and 8 months after relapse, but neither achieved remission. Before starting rituximab treatment, these two patients had been discontinued from immunosuppressive therapy for more than three months and were being treated with Chinese herbal medicine. However, nephrotic syndrome had not resolved under this treatment regimen. All patients had used renin-angiotensin-system inhibitors and 11 had used them for more than three months before rituximab treatment. Frequent or important comorbidities at baseline included hypertension (*n* = 19), diabetes (*n* = 4), coronary heart disease (*n* = 1), chronic obstructive pulmonary disease (*n* = 1), and hepatitis B virus carriers (*n* = 2). The values of the parameters at baseline were as follows: urinary protein, 8.5 ± 3.6 g/day; serum ALB, 24.8 ± 3.4 g/L; and eGFR, 88 ± 25 mL/min/1.73 m^2^. There were 18 patients in CKD1 stage, 11 patients in CKD2 stage, and 3 patients in CKD3 stage. The median follow-up period was 24 (18–38) months. Patients were divided into two groups based on the anti-PLA2R titer, viz. the low-titer group (anti-PLA2R titer < 150 RU/mL, *n* = 17) and high-titer group (anti-PLA2R titer ≥ 150 RU/mL, *n* = 15). There were no significant differences in sex, age, urinary protein, serum ALB, or eGFR levels between the two groups at baseline (*p* > 0.05).

### B-cell depletion

**Fig. 2:**
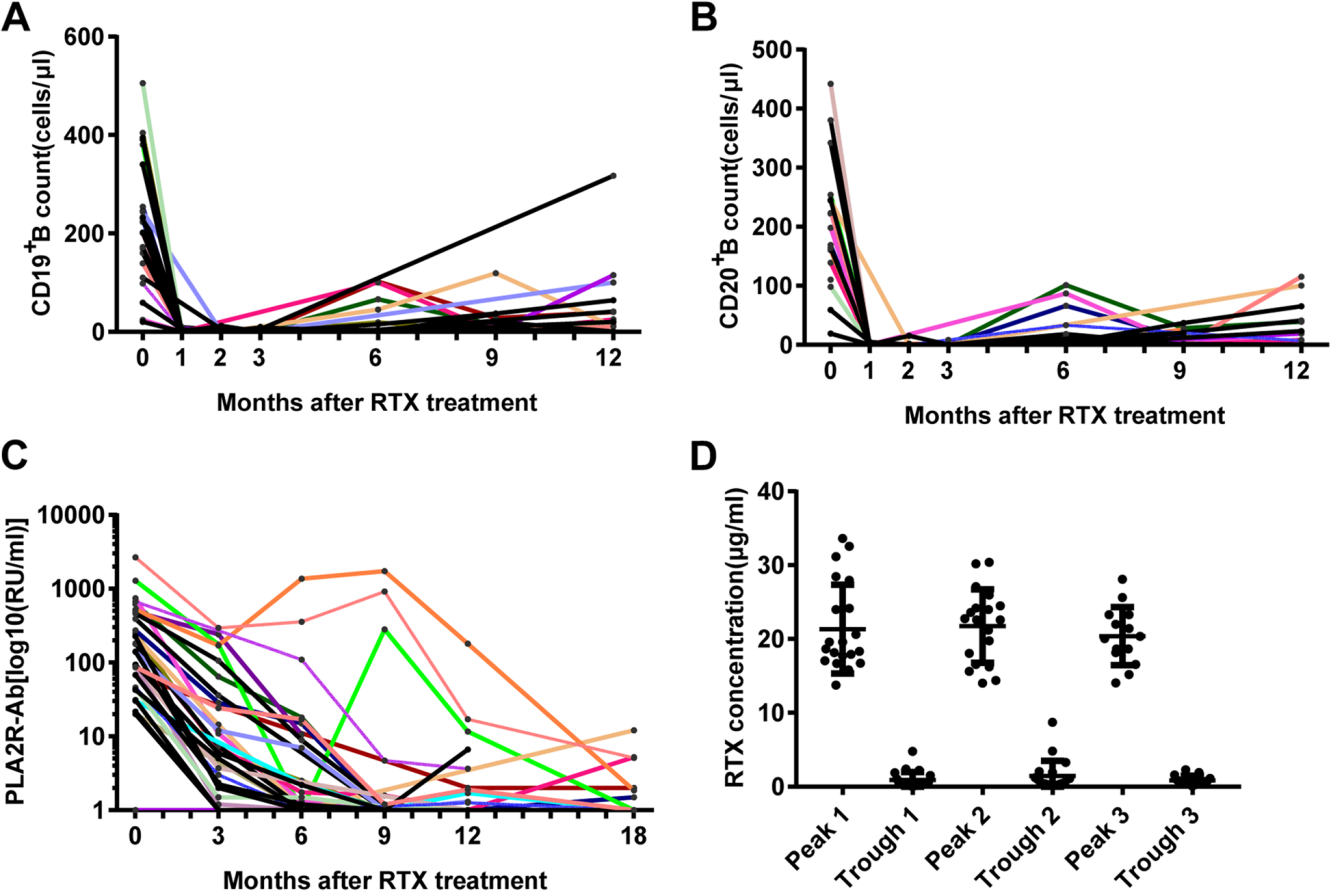
Immunological changes in individual patients before and after monthly mini-dose rituximab treatment. **A**. CD19 + B lymphocyte count; **B**. CD20 + B lymphocyte count; **C**. Anti-phospholipase A2 receptor antibody titer; **D**. Circulating peak and trough rituximab concentration(Peak: samples were obtained the next morning after rituximab infusion; trough: samples were acquired a couple of days before the next rituximab treatment)

Our data showed that 87.5% (28/32) of patients achieved B-cell depletion before the second dose (one month after the first dose of rituximab 100 mg). All patients achieved B-cell depletion one month after the second dose of rituximab 100 mg (Fig. 2A-B). The B-cell count was maintained at < 5/mm^3^ as long as rituximab administration continued, and it recovered a few months after rituximab cessation. There was no significant difference in B-cell depletion between the two groups (Table 1).

**Table 1:** Baseline characteristics and follow-up data of the two groups

	All (*N* = 32)	Low anti-PLA2R group (*n* = 17)	High anti-PLA2Rgroup (*n* = 15)	*P* value
Age (years)	55 ± 15	52 ± 13	59 ± 16	0.178
Sex (M/F)	25/7	13/4	12/3	0.261
Baseline				
Urinary protein (g/24 h)	8.5 ± 3.6	8.1 ± 3.4	8.9 ± 3.9	0.509
Serum albumin (g/L)	24.8 ± 3.4	25.8 ± 2.9	23.6 ± 3.6	0.069
eGFR (mL/min/1.73m^2^)	88 ± 25	91 ± 24	85 ± 27	0.491
anti-PLA2R titer (RU/mL)	320 ± 511	62 ± 39	611 ± 637	0.005
CD19 + B cell (/mm^3^)	226 ± 128	211 ± 117	237 ± 141	0.642
Immunoglobulin G (g/L)	6.1 ± 2.5	5.4 ± 1.4	6.8 ± 3.1	0.109
6 months				
Urinary protein (g/24 h)	3.7 ± 3.0	3.4 ± 3.2	4.1 ± 2.9	0.530
Serum albumin (g/L)	32.2 ± 5.2	33.5 ± 4.5	30.8 ± 5.7	0.172
eGFR (mL/min/1.73m^2^)	85 ± 26	95 ± 25	74 ± 23	0.023
anti-PLA2R titer (RU/mL)	69 ± 264	4 ± 6	135 ± 367	0.203
CD19 + B cell (/mm^3^)	14 ± 30	21 ± 40	8 ± 15	0.294
Immunoglobulin G (g/L)	7.7 ± 2.4	7.3 ± 2.1	8.0 ± 2.6	0.507
rituximab cumulative dose (mg)	525 ± 139	476 ± 130	580 ± 108	0.021
12 months				
Urinary protein (g/24 h)	1.8 ± 2.1	1.0 ± 1.2	2.7 ± 2.5	0.028
Serum albumin (g/L)	36.7 ± 6.2	39.6 ± 3.9	33.4 ± 6.8	0.007
eGFR (mL/min/1.73m^2^)	82 ± 25	88 ± 24	74 ± 25	0.115
anti-PLA2R titer (RU/ml)	8 ± 33	1 ± 2	17 ± 49	0.279
CD19 + B cell (/mm^3^)	33 ± 70	48 ± 89	14 ± 22	0.246
Immunoglobulin G (g/L)	9.9 ± 3.2	10.6 ± 3.3	9.1 ± 3.0	0.298
rituximab cumulative dose (mg)	747 ± 253	659 ± 255	847 ± 217	0.033
PR + CR, n (%)	25 (78)	16 (94)	9 (60)	0.020
CR, n (%)	8 (25)	6 (35)	2 (6)	0.152
18 months				
Urinary protein (g/24 h)	1.8 ± 3.0	1.4 ± 3.7	2.2 ± 1.8	0.445
Serum albumin (g/L)	39.3 ± 5.7	41.3 ± 5.4	37.0 ± 5.4	0.033
eGFR (mL/min/1.73m^2^)	79 ± 21	84 ± 19	74 ± 22	0.274
anti-PLA2R titer (RU/ml)	2 ± 3	1 ± 1	3 ± 4	0.092
rituximab cumulative dose (mg)	819 ± 351	694 ± 270	960 ± 387	0.030
PR + CR, n (%)	27 (84)	16 (94)	11 (73)	0.106
CR, n (%)	11 (34)	9 (53)	2 (13)	0.000
B-cell depletion				
Time for B cell depletion (m)	1.1 ± 0.4	1.1 ± 0.4	1.1 ± 0.4	0.994
rituximab dose for B cell depletion (mg)	113 ± 34	112 ± 33	107 ± 26	0.898
Time to anti-PLA2R titer decrease (m)				
≥ 50%	1.4 ± 0.8	1.1 ± 0.5	1.7 ± 1.0	0.062
≥ 90%	4.1 ± 3.5	3.5 ± 3.4	4.8 ± 3.5	0.308
< 2 (RU/mL)	7.0 ± 5.0	5.8 ± 5.1	8.8 ± 4.6	0.126
rituximab cumulative dose for anti-PLA2R titer decrease (mg)				
≥ 50%	137 ± 55	124 ± 44	153 ± 64	0.142
≥ 90%	341 ± 251	253 ± 128	440 ± 318	0.033
< 2 (RU/mL)	507 ± 362	335 ± 169	750 ± 427	0.007

Mean Â ± standard deviation (SD) Abbreviations: *eGFR *estimated glomerular filtration rate, *anti-PLA2R *anti-phospholipase A2 receptor antibody, *PR *Partial remission, *CR *Complete remission

### Anti-PLA2R titer

The baseline anti-PLA2R concentrations in the low- and high-titer groups were 62 ± 39 and 611 ± 637 RU/mL, respectively (*p* = 0.005). Most individuals showed a progressive decline after two doses of rituximab (Fig. 2C). All but one patient in the low-titer group and 66.7% (10/15) of patients in the high-titer group exhibited anti-PLA2R titers ˂ 2 RU/mL by 12 months. The high-titer group required a greater number of rituximab doses (750 ± 427 vs. 335 ± 169 mg; *p* = 0.007) to attain negative conversion of anti-PLA2R (Table 1) compared to the low-titer group, owing to the same declining trend.

2A. CD19 + B lymphocyte count; 2B. CD20 + B lymphocyte count; 2C. Anti-phospholipase A2 receptor antibody titer; 2D. Circulating peak and trough rituximab concentration(Peak: samples were obtained the next morning after rituximab infusion; trough: samples were acquired a couple of days before the next rituximab treatment).

### Clinical remission of NS

**Fig. 3:**
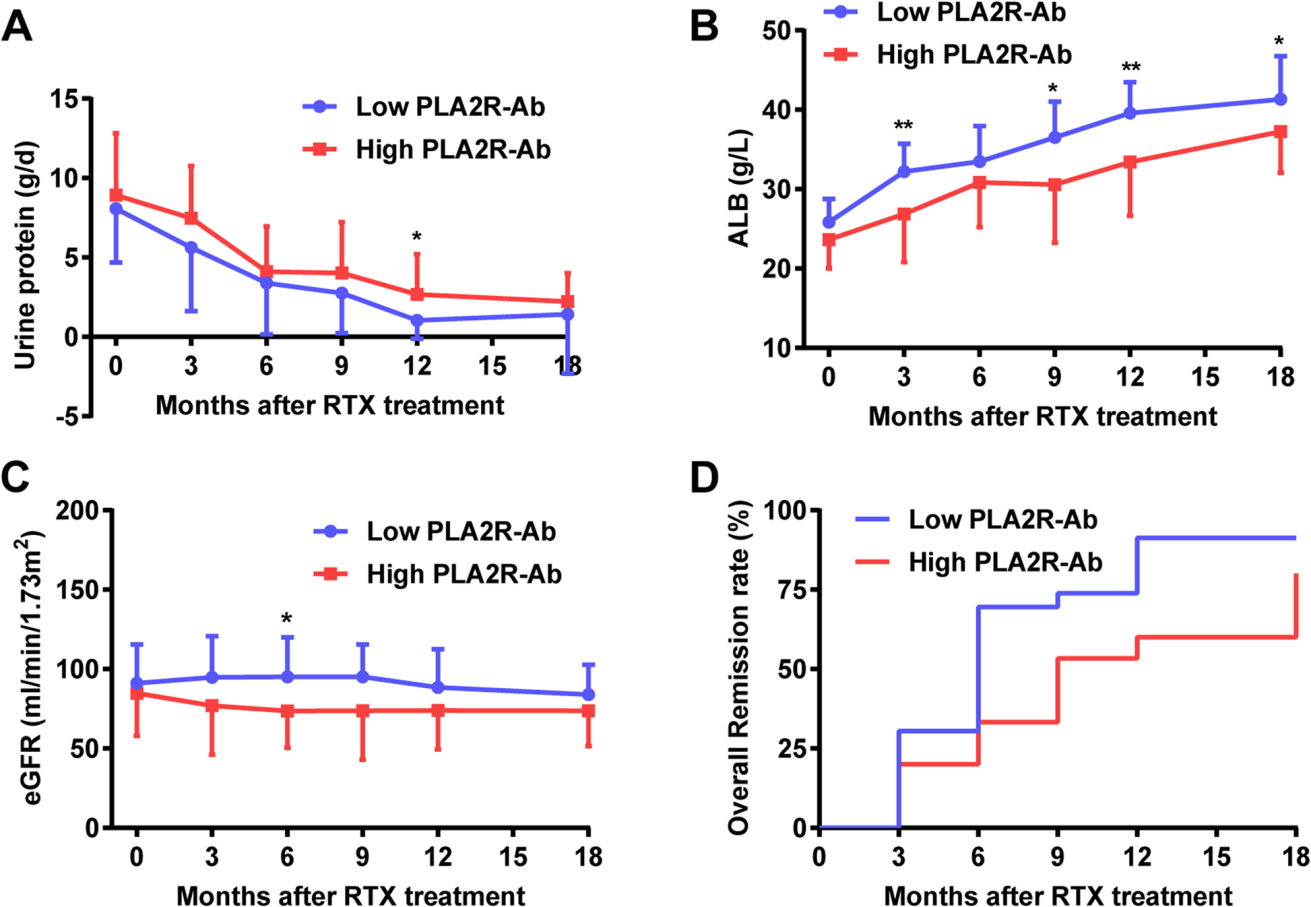
Comparison of the clinical data between different anti-PLA2R titer groups before and after monthly mini-dose rituximab treatment. **A**. Decrease in urinary protein excretion; **B**. Increase in serum albumin; **C**. Changes in the estimated glomerular filtration rate; **D**. Overall remission rate. Blue line: low anti-PLA2R titer group, red line: high anti-PLA2R titer group. PLA2R: phospholipase A2 receptor. *p < 0.05, ** *p* < 0.01

The urinary protein levels decreased and serum ALB levels increased gradually after rituximab treatment (Fig. 3A-B). The remission rate at six months was 46.9% (15 patients) with a CR rate of 6.3% (2 patients), while that at 12 months was 78.1% (25 patients) with a 25% CR rate (8 patients), and that at 18 months was 84.3% (27 patients) with a 34.3% CR rate (11 patients). By 18 months, the urinary protein (*p* = 0.445, Fig. 3A), eGFR (*p* = 0.115, Fig. 3C) and the clinical remission rate (73% vs 94%, *p* = 0.106, Fig. 3D) were not significantly different between the two groups. In contrast, the cumulative dose of rituximab (960 ± 387 vs 694 ± 270 mg, *p* = 0.030) was higher in the high-titer group than in the low-titer group, while serum ALB (37.0 ± 5.4 vs 41.3 ± 5.4 g/L, *p* = 0.033, Fig. 3B) and the complete remission rate (13% vs 53%, *p* = 0.000) were both lower in the high-titer group than in the low-titer group. The anti-PLA2R antibody concentration was ˂ 2 RU/mL in all but one patient who achieved clinical remission, but not all patients with anti-PLA2R antibodies ˂ 2 RU/mL achieved clinical remission.

The relapse rates at 12 months, 18 months, and last follow-up were 0, 3%, and 6%, respectively. Two patients relapsed. One case occurred at 17 months of follow-up and 6 months after the last rituximab infusion. The other case occurred at 21 months of follow-up and 11 months after the last infusion. The median relapse-free survival from the last infusion was 13.5 months (range 3–27).

### Peak and trough concentration of circulating rituximab

The peak and trough concentrations of rituximab were measured thrice at different treatment cycles for all patients (Fig. 2D). The peak rituximab samples were obtained on the next morning after rituximab infusion. The three peak rituximab concentrations were 21.3 ± 6.0, 21.8 ± 5.0, and 20.4 ± 3.9 µg/mL, which did not exhibit any statistically significant difference (*p* > 0.05). The trough rituximab samples were acquired a couple of days before the next rituximab treatment. The three trough concentrations were 0.9 ± 1.1, 1.4 ± 2.1, and 0.9 ± 0.6 µg/mL respectively for all patients, which did not exhibit any statistically significant difference (*p* > 0.05). From the peak and trough rituximab concentration fluctuations, we concluded that each rituximab dose of 100 mg was exhausted in one month and additional administration was needed to raise the serum level of rituximab to an effective concentration.

### Adverse reactions

Seven instances of mild infusion reactions occurred for a total of 241 injections. Eight episodes of infection occurred in 6 patients. All patients recovered soon after receiving antibiotics. No other serious adverse reactions were observed.

## Discussion

Our study shows that monthly mini-dose rituximab monotherapy was successful in treating PLA2R-associated PMN. The NS remission rate after eighteen months was 84% at an average dose of rituximab 820 mg (range, 300–1800 mg). Clinical remission rates were similar in both groups, but the anti-PLA2R high-titer group required more cumulative doses of rituximab than the low-titer group.

Rapid and long-lasting B-cell depletion is essential to achieve a good therapeutic effect with B cell-targeting treatments. We set out to investigate whether rituximab 100 mg could achieve rapid B-cell depletion. Our data show that a single dose of rituximab 100 mg could achieve B-cell depletion in 87% of individuals, and that such depletion could be maintained for at least one month. We speculated that the rationale underlying this observation was as follows. First, the B-cell count in peripheral blood was within the normal range in patients with PMN. Therefore, a lower dose of rituximab was needed to achieve B-cell exhaustion compared to lymphoma. Ramachandran et al. also achieved CD19 depletion with a single dose of rituximab 100 mg [13]. Literature confirmed CD19 + B-cell depletion occurs fast (within few hours) and almost all patients achieve it, even after receiving small doses [14]. Therefore, a “CD19-targeted therapy” has been proposed to avoid unnecessary additional infusions and prevent relapses when needed [14].

Long-lasting B-cell depletion is beneficial for the negative conversion of anti-PLA2R antibodies, and can be accomplished by a regular dose of rituximab with a high peak, resulting in a strong depletion of B cells initially, followed by a gradual decline [15]. However, we adopted a different approach by administering rituximab monthly. Seitz-Polski et al. compared the NICE and GEMRITUX studies and found that the initial frequency of rituximab administration and remission rate were higher in the former (1 g on days 1 and 15). They found that the residual rituximab level after 3 months was greater in the NICE cohort than that in the GEMRITUX cohort, which is an important factor impacting the choice of dosing regimen. Three months after administration, the serum rituximab concentration was measurable in about half of the patients in the NICE study, but undetectable in almost all patients in the GEMRITUX study (rituximab 375/m^2^on days 1 and 8) [16]. Our regimen of monthly administration of rituximab 100 mg guaranteed a more effective rituximab concentration compared to the expected residual rituximab level at 3 months.

We think that a lower degree of loss via urine was probably another advantage of our mini-dose rituximab regimen. The pharmacokinetics of rituximab in PMN is unknown; some studies report that it differs substantially from that of follicular lymphoma and other autoimmune diseases [15, 17]. The half-life of rituximab is shorter in PMN (approximately 11.5 days) than in lymphoma (approximately 20 days) [15], probably because of the gross loss in urine [18]. Rituximab has been shown to be detectable in the urine of patients with PMN. This loss via urine caused the residual rituximab levels at month 3 to be significantly lower in PMN patients compared to myasthenia gravis patients with no proteinuria (matched for age, gender, and weight, and treated with a similar treatment regimen) [17]. Although we did not detect rituximab lost in the urine, based on the above literature there is reason to speculate that the standard rituximab protocol would have resulted in more loss via urine during the gross proteinuria period, whereas our mini-dose rituximab regimen resulted in less loss, which declined even further in the following months because of the amelioration of proteinuria.

The minimum effective dose of rituximab required for B-cell depletion is not currently known with precision, but it warrants discussion. Considering the peak concentration observed in our study, we speculated that rituximab 20 + µg/mL approximated the minimum effective serum concentration. There is some evidence to support this speculation. First, it was demonstrated to be effective in our study. Second, Iijima et al. reported that almost no recurrence occurred after 3 months with an average serum rituximab concentration of 28.8 µg/mL in steroid-resistant children with nephrotic syndrome (minimal change disease or focal segmental glomerulosclerosis) [19]. Third, the residual concentration of rituximab (approximately 20 + µg/mL) at three months contributed to the higher remission rate in the NICE cohort compared to undetectable residual rituximab at three months with a lower remission rate in the GEMRITUX study [16]. These data strengthen our claim that rituximab 20 + µg/mL is an effective serum concentration, and may approximate the minimal effective dose of rituximab.

We were unsure about the suitable interval for next dose after the initial administration of rituximab 100 mg. Based on the monthly cyclophosphamide experience, we administered rituximab 100 mg at monthly intervals. One month after rituximab 100 mg administration, the average trough rituximab concentration was 1.0 µg/mL, and the B cells were still in a state of exhaustion. We deliberated whether an additional dose of rituximab should be given at that time, or if it was more appropriate to wait for B-cell replenishment. We could not find any study that utilized a similar rituximab concentration in patients with PMN, although one study that investigated minimal change disease reported that the rate of recurrence at 3 months was significant when the average rituximab concentration was 2.3 µg/mL [19]. Thus, we thought that the trough concentration of rituximab in our study was not effective and that supplementation was necessary, despite the persistence of B-cell exhaustion. If the pathogenic factors causing PMN persist, B-cell regeneration may lead to a resurgence or elevation in anti-PLA2R antibodies, which would be remedied by monthly rituximab supplementation. Although the metabolism of rituximab in patients with PMN is incompletely understood, a monthly intermittent mini-dose regimen could maintain the rituximab concentration between the peak-to-trough fluctuations; moreover, continuous B-cell depletion was observed, which blocked anti-PLA2R antibody production, eventually achieving an immunological target response of ≤ 2 RU/mL.

The NS remission rate with the mini-dose rituximab regimen was non-inferior to the regular rituximab strategy. Our remission rate was 46.9% with a 6.3% CR rate at six months, which is not inferior to the results of previous studies. The six-month remission rates in the renowned randomized controlled trials (RCTs) were as follows: 35% in the GEMRITUX study [4], 35% in the MENTOR study [5], 44% in the STARMEN study [6], and 51% in the RI-CYCLO study [7]. Our remission rate was 78% with 25% CR at twelve months (average cumulative dose of rituximab 750 mg), which was also non-inferior to the results of previous studies. The twelve-month remission rates were 60% in the Mentor study [5], 51% in the STARMEN study [6], and 62% in the RI-CYCLO study [7]. Ramachandran et al. reported 50% remission in six patients with PLA2R-related refractory PMN with 2–4 doses of rituximab 100 mg at six months, [13] which bore close resemblance to our results.

Single or multiple infusion strategies for rituximab 100 mg, which yielded positive results, have been reported for other autoimmune diseases, including ABO-incompatible living-donor kidney transplantation [20], de novo donor-specific anti-HLA antibody-associated renal transplantation [21], steroid-dependent minimal change NS [22], and steroid-refractory thrombocytopenia due to systemic lupus erythematous [23], Our treatment course was relatively flexible and included cumulative doses and dosing intervals. The cumulative doses varied individually according to the anti-PLA2R levels and remission. In fact, the high anti-PLA2R group required higher cumulative rituximab doses and longer treatment durations. The dosing intervals were not very strict; a delay or advance of one or two weeks was acceptable. There was a gap of up to 2–3 months in the treatment regimens of some patients due to the COVID-19 epidemic and control measures in China, visible/potential infection, or other events that were deemed more important than PMN.

A progressive decline in serum anti-PLA2R antibodies after two doses of rituximab was observed in most individuals in our study. Since well-differentiated plasma cells may continue producing anti-PLA2R antibodies, the serum anti-PLA2R antibody levels may continue to rise even after the first rituximab dose, and decrease only upon exhaustion of the existing plasma cells. This steady decline was indicative of the onset of the effect of rituximab. As long as the B cells were in a state of depletion, the decline in the anti-PLA2R antibody levels was sustained. The rate of decline of the anti-PLA2R antibodies was similar; thus, the low-titer group was more likely to reach the target of ˂ 2 RU/mL than the high-titer group, which means that a higher dose of rituximab would be needed in the latter.

Recurrence is a concern because of the small total amount of rituximab administered in our study. However, the median follow-up duration was 24 months (range, 18–38), and two recurrences were observed. The relapse rate in the rituximab group in the STARMEN trial was 7% (3/43), and that in the RI-CYCLO study was 8% (3/37), which were very similar to our cohort. This may be attributed to frequent administration and longer total effective rituximab period.

Rituximab is a B cell-targeted therapy. It has already been recommended as first-line treatment for PMN by the 2021 KDIGO guidelines [2]. The guidelines recommend two standard treatment regimens for rituximab; however, clinically, there is controversy regarding the specific dosage and interval. Fenoglio et al. reported that the effects of rituximab 375 mg/m^2^administered once and four times were the same when treating PMN [24]. We believe that the specific dosing regimen should be adjusted according to the patient’s age, primary disease, comorbidities, and immune status. Mini-dose rituximab may be more suitable for the “vulnerable” subset of the PMN population (such as the elderly, patients susceptible to infection, those who have newly recovered from a severe infection, or patients with very low serum immunoglobulin, and low anti-PLA2R antibody titer). The regimen should not be adopted in PMN patients with high anti-PLA2R titer. The KDIGO guidelines consider an anti-PLA2R antibody > 50 RU/mL to be among the additional conditions for high risk of recurrence. Previous studies have shown that patients with high titers of PLA2R antibodies have low rates of spontaneous remission [25]. Therefore, we inferred that patients of PMN with high titers of anti-PLA2R antibodies treated with optimal supportive care and observation for 6 months are not optimal candidates for the mini-dose regimen. Immediate initiation of immunosuppressive therapy in conjunction with maximal supportive care is a rational regimen if clinicians consider the patient to be at a high or very high risk of progression to ESRD. From the perspective of health economics, rituximab is not covered by medical insurance in China. We achieved a non-inferior response rate compared to standard therapy using approximately one-fourth the conventional dose, which also reduced the medical cost. Our mini-dose, frequent-administration regimen makes rituximab affordable for patients with limited financial resources.

We were also concerned that continuous maintenance of B-cell depletion may affect normal immunoglobulin expression and lead to persistent humoral immune dysfunction [9, 10]. However, our data showed that immunoglobulin G levels at six and twelve months were significantly higher than those at baseline. Moreover, serum immunoglobulin G levels in the remission group were significantly higher than those in the non-remission group at six and twelve months, which suggested that the faster the remission of NS, the earlier the recovery of humoral immune function. A total of eight episodes of infections occurred in six patients in our cohort (19%), all of whom recovered rapidly. This is comparable with other previously published papers on rituximab therapy in MN. The infection rates were 28% in the Mentor study [5], 3% in the GEMRITUX Study [4], 30% in the STARMEN study [6], and 14% in the RI-CYCLO study [7].

Another potential concern is the development of anti-rituximab antibodies with this mini-dose repeated regimen. Patients with autoimmune disorders exhibit greater risk of anti-rituximab antibody development and anti-rituximab antibody-related adverse events [26]. Since the efficacy and safety of our mini-dose monthly therapy was similar to that of standard care, we did not check whether patients developed antibodies against rituximab or not.

Our study has some limitations. This real-world retrospective observational study was conducted at a single center with a small sample size. Another limitation was that only 10 of the 32 patients used the maximum tolerable dose of RASI for more than 6 months before starting rituximab therapy. According to KDIGO guidelines, there is a 20–45% chance of spontaneous remission in membranous nephropathy, and consequently, starting immunosuppressive therapy before 6 months of conservative therapy might overestimate the response rate to therapy [2]. Further multicenter RCTs comparing low-dose rituximab with standard doses of rituximab are needed to confirm the efficacy and safety of this strategy.

In summary, our data suggest that monthly rituximab 100 mg appeared as a potential effective regimen for treating anti-PLA2R-associated primary membranous nephropathy with a low anti-PLA2R titer, especially suitable for a certain subset of patients, such as the elderly, those susceptible to infection, and patients with limited financial resources. However, this regimen should not be adopted in PMN patients with high anti-PLA2R titer.

## Data Availability

The raw datasets analyzed in this study are available from the corresponding author upon reasonable request and with permission from the institutional review board.

## Abbreviations

ALB: Albumin
anti-PLA2R: Anti-phospholipase A2 receptor
COVID-19: Corona Virus Disease 2019
CR: Complete remission
eGFR: Estimated glomerular filtration rate
ESRD: End-stage renal disease
GEMRITUX: Evaluate Rituximab Treatment for Idiopathic Membranous Nephropathy
KDIGO: Kidney Disease: Improving Global Outcomes
MENTOR: Membranous Nephropathy Trial of Rituximab
NICE: Primary membranous nephropathy in the Department of Nephrology at Pasteur Hospital in Nice
NS: Nephrotic syndrome
PMN: Primary membranous nephropathy
PR: Partial remission
RCT: Randomized controlled trials
RI-CYCLO: Rituximab versus Steroids and Cyclophosphamide in the Treatment of Idiopathic Membranous Nephropathy
STARMEN: Sequential treatment with TAcrolimus-Rituximab versus steroids plus cyclophosphamide in patients with primary MEmbranous Nephropathy
